# Impact Modifiers and Compatibilizers for Versatile Epoxy-Based Adhesive Films with Curing and Deoxidizing Capabilities

**DOI:** 10.3390/polym13071129

**Published:** 2021-04-02

**Authors:** Bo-Young Lee, Dae-Hyeon Lee, Keon-Soo Jang

**Affiliations:** Department of Polymer Engineering, School of Chemical and Materials Engineering, The University of Suwon, Hwaseong 18323, Korea; bylee_96@naver.com (B.-Y.L.); rpdh8096@naver.com (D.-H.L.)

**Keywords:** epoxy, adhesive film, deoxidizing, impact modifier, compatibilizer, amorphous polymer

## Abstract

Epoxy resins with acidic compounds feature adhesion, robustness, and deoxidizing ability. In this study, hybrid adhesive films with deoxidizing and curing capabilities for semiconductor packaging were fabricated. The compatibilizing effects and mechanical properties were chiefly investigated by using various additive binders (thermoplastic amorphous polymers) and compatibilizing agents. The curing, deoxidizing, thermal, and rheological properties were systematically investigated. For uniform film formation and maximizing deoxidizing curable abilities, a thermoplastic–thermoset mixture containing a phenyl and carboxylic acid-based additive (benzoic acid), and a polycarbonate was chosen as the model adhesive film. Without either a phenyl or an acidic group in the compatibilizing agent, deoxidizing and compatibilizing effects were not achieved. The manufactured hybrid adhesive film can be effectively used, especially for electronic devices that require deoxidization and adhesion.

## 1. Introduction

Epoxy resins have been widely utilized in a myriad of applications, such as electronic and semiconductor packaging, owing to their excellent adhesion to various substrates, and their mechanical and thermal properties [1,2,3]. As an example, they have been used as structural adhesives for epoxy molding compounds (EMCs) which protect semiconductor chips from external environmental factors, such as impact, pressure, moisture, heat, and ultraviolet rays [4,5]. 

Epoxy resins have also been used as underfill substances that are filled between the chips and substrates and thus act as an underfill encapsulant as well as an adhesive [2,6,7]. For electrical interconnections between chips and substrates, Sn-based fusible metal solders, such as Sn/Ag and Sn/Ag/Cu caps on Cu pillars, have been utilized [8,9,10]. At elevated temperatures, the metal solders are melted and wetted, thereby creating an electrical path between the chips and substrates [11]. However, Sn-based metal solders are susceptible to oxidation on the surface in an ambient atmosphere [12,13,14]. Therefore, a reductant for removing an oxidized metal layer is required; this results in wetting and generation of the electrical path [15].

Electrical interconnections between chips and substrates are commonly achieved by melting and interconnecting solders (soldering) using deoxidizing flux materials at high temperatures (greater than the melting point of solder) prior to the additional process: that is, the epoxy underfill encapsulant process [16]. To overcome the tedious two-step procedure, a new soldering/underfilling process has been developed. The electrical interconnections were facilely achieved by an in situ bonding procedure using curable deoxidizing epoxy-based adhesives [11,15]. Hydroxyl, amino and acidic moieties, organoamine hydrochlorides, rosin-based fluxes, and inorganic acids in the state-of-the-art adhesives (known as no-flow underfill or fluxing underfill) remove the oxidizing layer of the metal pad (electrode) on the chips or substrates, thereby leading to the electrical interconnection via a melting and wetting of solders at elevated temperatures. Subsequently, the functional adhesives are completely cured, despite the presence of some residual deoxidizing additives in the resin [15].

The residues, such as reductants and additives, remain in the adhesives after complete curing, thus causing negative plasticization effects that reduce the thermal and mechanical properties of the functional adhesives [15,17]. Recently, anhydride-based adhesives endowed with curable deoxidizing capabilities without the deoxidizing additives (flux) were reported, thereby reducing the probability of residual substances [15].

In addition to the adhesive pastes, versatile epoxy/phenoxy/anhydride-based hybrid adhesive films were fabricated by combining thermosetting resins and a thermoplastic matrix [18]. The hybrid adhesive films can be facilely applied to substrates with the required dimensions and contents. The films were melted and the metal oxidized layers were removed at elevated temperatures to induce electrical interconnections. Finally, the adhesive films were networked between the chips and substrates. However, the mechanical and thermal properties of the cured adhesive films were not satisfactory. In this study, impact modifiers and compatibilizers were utilized to enhance the mechanical properties of functional adhesive films.

## 2. Experiment

### 2.1. Materials

#### 2.1.1. Polymer

**Figure 1:**
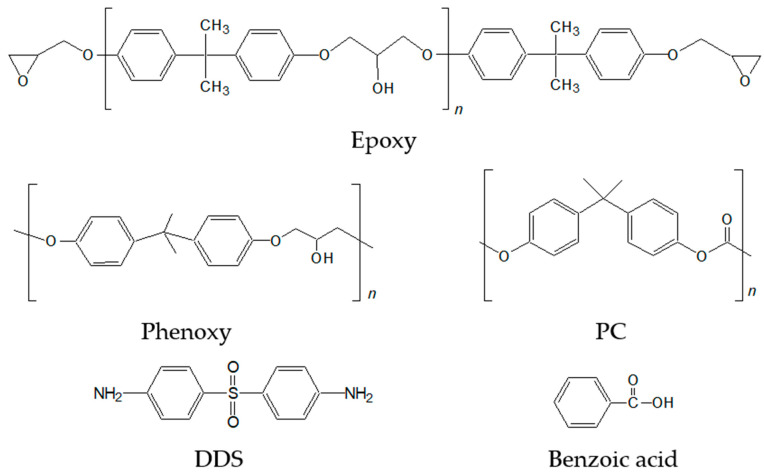
Chemical structures of epoxy, phenoxy, PC, DDS, and benzoic acid.

An epoxy binder (YD-128) containing a diglycidyl ether of bisphenol A (DGEBA) with an epoxy equivalent weight (EEW) of 187 g/eq and a phenoxy resin (YP-50) with a molecular weight of ca. 55,000 g/moL were supplied by Kukdo Chemicals Co. (Seoul, Korea). Polycarbonate (PC; Infino SC-1100R, Lotte Chemical Co., Seoul, Korea), poly(methyl methacrylate) (PMMA; IG840, LG MMA Co., Seoul, Korea), and polystyrene (PS; Styrolution PS 147F, BASF Co., Ludwigshafen, Germany) were utilized as impact modifiers. Their chemical structures are displayed in Figure 1 and Figure S1.

#### 2.1.2. Compatibilizer

Biphenyl (99.0%) and hexanoic acid (98.0%) were obtained from Tokyo Chemical Industry (TCI) Co. Ltd. (Tokyo, Japan). Benzoic acid (>99.0%), stearic acid and benzene (99.0%) were purchased from BNOChem Co. (Cheongju, Korea).

#### 2.1.3. Curing Agent and Solvent

Bis(4-aminophenyl sulfone) (DDS, >97.0%) were obtained from Tokyo Chemical Industry (TCI) Co. Ltd. (Tokyo, Japan). Dichloromethane (DCM, 99.8%) and 2-butanone (methyl ethyl ketone; MEK, 99.5%) were supplied by Samchun Pure Chemicals Co. (Seoul, Korea). 

#### 2.1.4. Metal Solder and Al Substrate

For wetting (soldering) tests, Sn-based metal solders (SAC305; 96.5% Sn/3.0% Ag/0.5% Cu) with a diameter of 700 µm and a melting point of 219 °C were purchased from Poongsan Co. (Seoul, Korea). Aluminum (Al) alloy (Al6061, Al: Mg: Si: Fe: Cu: Cr = ca. 97.2: 1.0: 0.6: 0.35: 0.25: 0.25 by mass, less than 0.35 wt% Zn/Ti/Mn) sheets with dimensions of 24 mm × 85 mm × 1.96 mm were supplied by Jun Tech Co. (Hwaseong, Korea).

### 2.2. Fabrication of Hybrid Adhesive Films

A thermoplastic-based matrix (phenoxy, PS, PMMA, and PC) (60 wt.%) was mixed in DCM using a vortex mixer (MX-S, DLAB Scientific Inc., Beijing, China) at room temperature for 1 h [18]. The EEW ratios between DGEBA and DDS were 1:1. The concentration of benzoic acid was varied between 0 and 10 phr. DDS and benzoic acid were mixed in MEK for 3 min. Then, the solution was added to the epoxy binder and mixed for 3 min using the vortex mixer. Subsequently, the 40 wt.% epoxy resin (DGEBA-based binder and curing agent) and the 0–10 phr deoxidizing agent (benzoic acid) were added to the thermoplastic blend solutions and mixed for 3 min at room temperature. The viscosity of the mixture solution was controlled by drying the solution at room temperature. The mixture was then poured onto a silicone substrate. The thickness of the mixture (0.5 mm) was controlled via a doctor blade method and then it was completely dried at room temperature for 1 h to form a 100 µm adhesive film. The sample names are abbreviated and listed in Table 1. For example, a hybrid film containing 40 wt.% 1:1 epoxy/DDS (E/D), 55 wt.% phenoxy (P), 5 wt.% PC (C), and 5 phr benzoic acid (**Ba**) was denoted as EDPC5**Ba5**.

### 2.3. Adhesive Film Bonding Procedure

Aluminum specimens were cleaned in acetone via an ultrasonic cleaner for 20 min prior to bonding. An adhesive film with dimensions of 24 mm × 10 mm was placed between the Al substrates. Subsequently, a weight of 200 g was applied to the sandwiched samples and then the adhesive film was cured at 250 °C for 5 min.

### 2.4. Characterization

Differential scanning calorimetry (DSC; Model DSC25, TA Instruments Inc., New Castle, DE, USA) was conducted to monitor the curing behaviors of the hybrid adhesive films at a scanning rate of 10 °C/min under nitrogen purging (50 mL/min). Approximately 3 mg of the hybrid adhesive film was placed in a hermetic aluminum DSC pan.

Fourier transform infrared (FTIR) spectroscopy (Spectrum Two, PerkinElmer Inc., Waltham, MA, USA) with attenuated total reflection (ATR) mode was utilized to detect the epoxide, ether, amino and acidic groups of the adhesive films before and after curing. Each spectrum at 4000–400 cm^−1^ was obtained using a KBr pallet with a scan number of 16. The samples used for ATR FTIR spectroscopy were cured at 100 °C for 30 min, at 150 °C for 30 min, and then at 200 °C for 1 h.

For the wetting tests [19,20,21], six metal solder balls were placed between the Cu plate and the uncured hybrid adhesive films as shown in Figure S2a. The wetting tests were performed for potential application to the bonding process between chips and substrates (Figure S2b). The sample was heated on a hot plate at 250 °C in air. The initial wetting time and the number of wetted metal solder balls were recorded.

A universal testing machine (UTM; LR10K Plus, Lloyd Instruments, AMETEK Inc., Berwyn, PA, USA) was employed to measure the shear strength and toughness of the cured adhesive films between Al substrates at a crosshead rate of 1 mm/min. The toughness values were determined from the integrated areas of the resulting stress-strain curves.

Scanning electron microscopy (SEM; Apreo, FEI Co., Hillsboro, OR, USA) at the Center for Advanced Materials Analysis was utilized to examine the morphologies of the fractured cured samples. The samples on the carbon tape for SEM examination were sputter-coated with Au.

A torsional parallel plate rheometer (MCR 300, Anton Paar, Graz, Austria) was utilized to investigate the rheological properties of the uncured adhesive films. The viscosities of the adhesive films were measured under dynamic conditions at a frequency of 1 Hz and a controlled strain (displacement mode) of γ = 0.01. The heating rate was 3 °C/min. 

## 3. Results and Discussion

**Figure 2:**
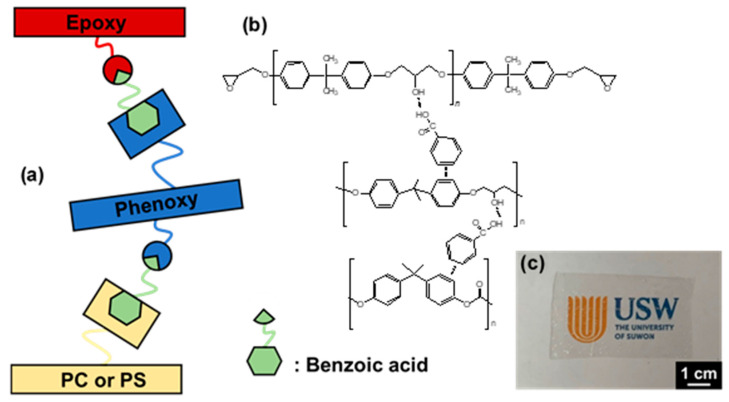
(**a**,**b**) Compatibilizing mechanism of hybrid adhesive films and (**c**) manufactured hybrid film of EDPC5**Ba5**.

The mechanical properties of adhesive materials in semiconductor packaging are crucial because electronic devices are exposed to various extreme conditions. Thus, we examined the mechanics as well as the curing, deoxidizing, thermal, and rheological properties by using amorphous polymer additives and compatibilizing agents (Figure 2) with curing and deoxidizing capabilities.

**Figure 3:**
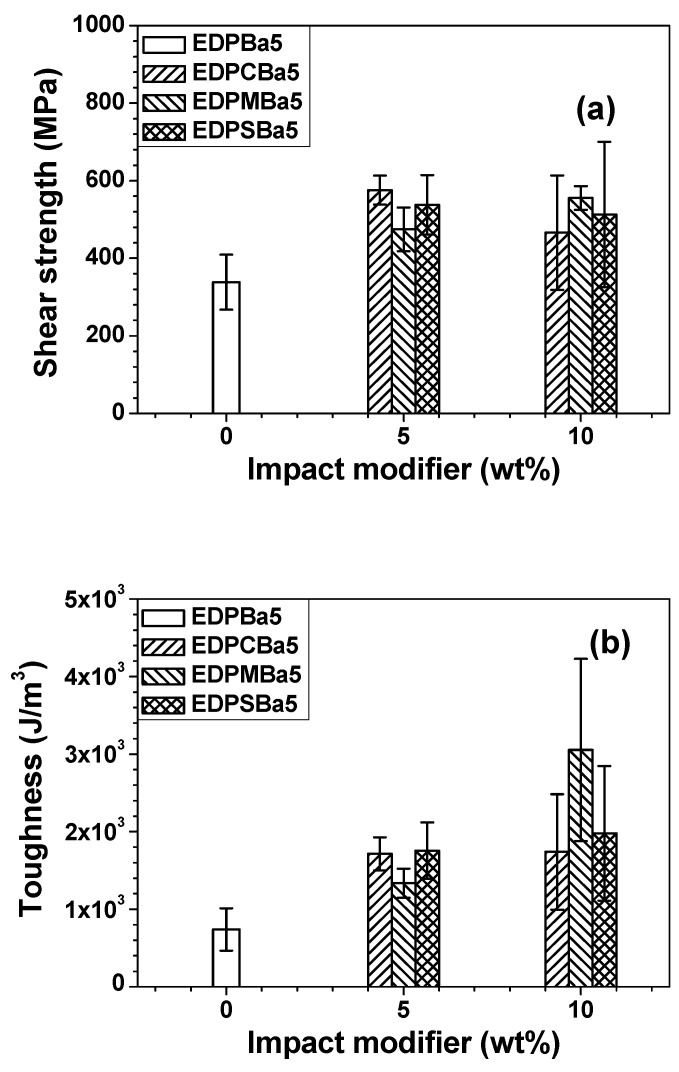
Shear strength (**a**) and toughness (**b**) of adhesive films incorporated with PC, PMMA, or PS.

**Figure 4:**
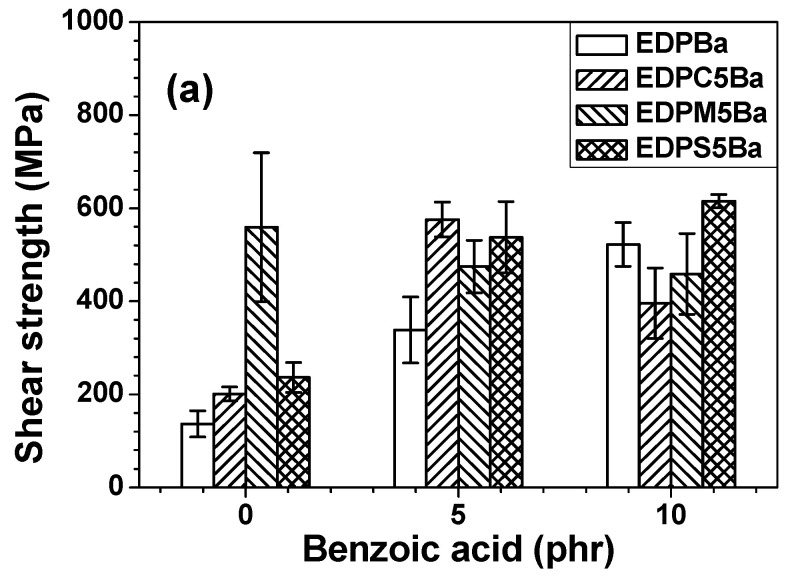

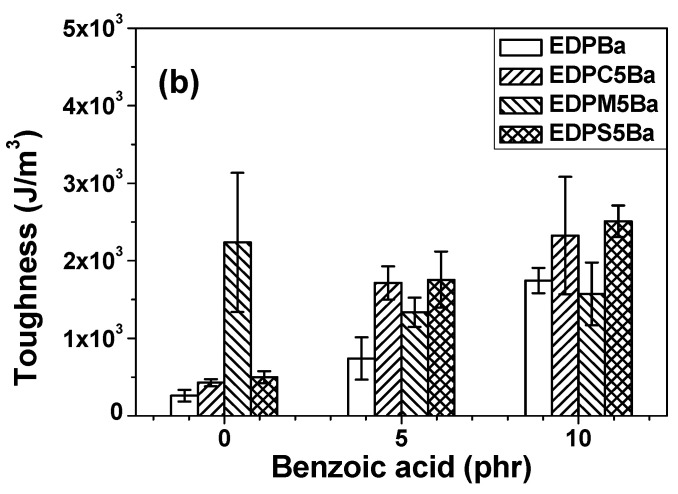
(**a**) Shear strength and (**b**) toughness of adhesive films containing PC, PMMA, or PS as a function of **Ba**.

Figure 3 shows that the incorporation of binders (PC, PMMA and PS) into the EDP hybrid adhesive film composed of the primary epoxy binder, amine curing agent, and phenoxy matrix improved both its shear strength and toughness. However, above 10 phr PC or 10 phr PS, the shear strength was reduced, probably as a result of the uncompatibilizing behaviors between the phenoxy main matrix and amorphous polymer additives. Polymers are commonly incompatible and immiscible with one another [22,23,24]. As shown in Figure 4, it was found that the compatibilizing agent (benzoic acid) reduced the interfacial energy among each component, thereby leading to compatibilizing effects. The shear strength and toughness of EDP-based adhesive films considerably increased when increasing the benzoic acid (**Ba**) concentration. The adhesive film without the PC, PMMA, and PS additives also exhibited enhanced mechanical properties. This indicates that the incorporation of **Ba** into the adhesive mixture resulted in the compatibilizing effects not only between the phenoxy resin and amorphous polymer additives but also between the phenoxy and epoxy resins. The mechanical properties of the EDPM**Ba** adhesive series containing PMMA (M) decreased as a function of **Ba** content. Morphological studies should be performed to examine the causes of the changes in mechanical properties.

**Figure 5:**
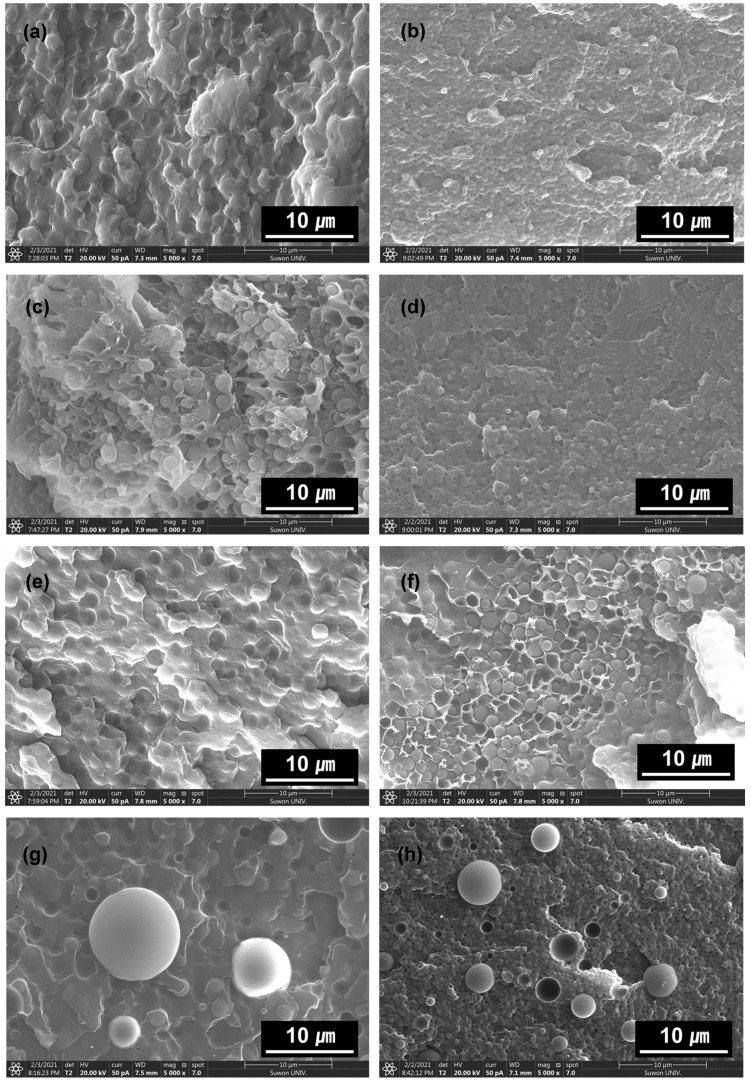
SEM images of fractured cured adhesive film: (**a**) EDP, (**b**) EDP**Ba5**, (**c**) EDPC5, (**d**) EDPC5**Ba5**, (**e**) EDPM5, (**f**) EDPM5**Ba5**, (**g**) EDPS5, and (**h**) EDPS5**Ba5**.

Visual observation of the phase morphologies for the polymer blends is routinely investigated by SEM. Figure 5 and Figure S3 show that the domain sizes in the matrices of EDP, EDPC5, and EDPS5 were reduced and compatibilized by the incorporation of 5 phr **Ba**. In contrast, the **Ba** in the EDPM5 adhesive films exhibited phase separation and incompatibility between PMMA and the phenoxy resin because of the structural relationship. PMMA has no aromatic rings or moieties that can form hydrogen bonds. Therefore, benzoic acid failed to compatibilize the blends but remained as a plasticizer and impurity, thereby decreasing the interfacial adhesion between the polymer domain and phenoxy/epoxy matrices. This morphological behavior examined by SEM was in good agreement with the mechanical property results.

**Figure 6:**
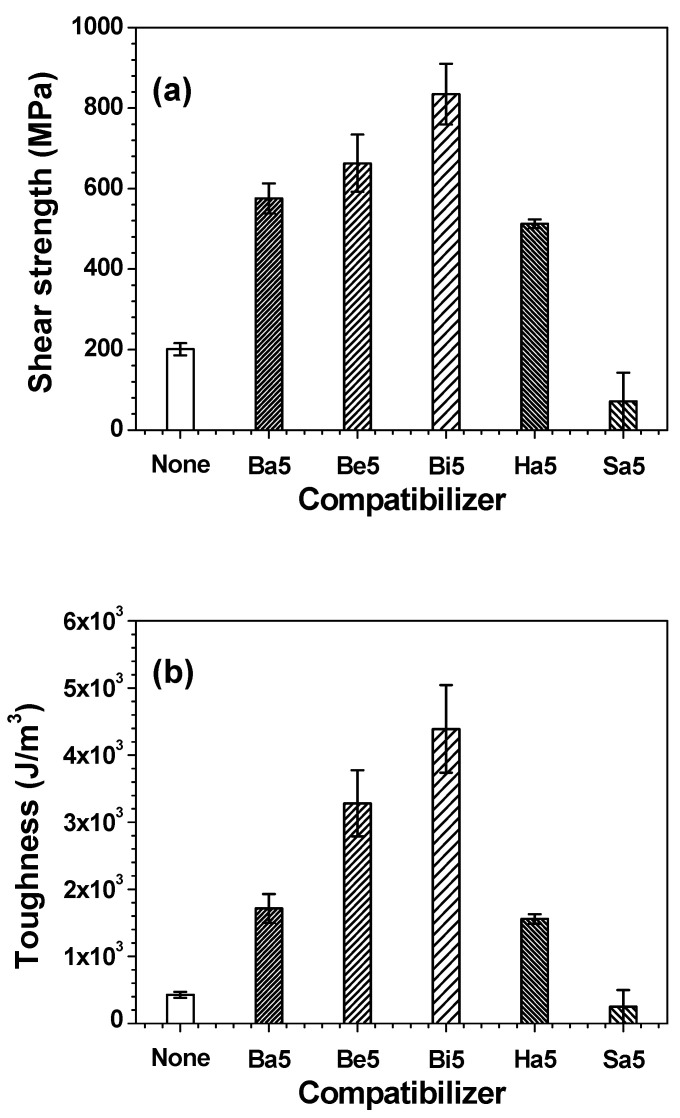
(**a**) Shear strength and (**b**) toughness of EDPC adhesive films with different compatibilizers.

Benzoic acid (**Ba**), benzene (**Be**), biphenyl (**Bi**), hexanoic acid (**Ha**), and stearic acid (**Sa**) were added to the EDPC adhesive films to further examine the structural interplay, as shown in Figure 6. For the formation of films, the EDPC5 series was selected based on the images in Figure S4 because, beyond 10 phr PC (C), the film morphologies became phase-separated with the loss of transparency. Among the various additives, the infiltration of 5 phr **Bi** into the EDPC5 adhesive film showed the highest compatibilizing effect by increasing the shear strength (×4; from 200 to 840 MPa) and toughness (×10; from 430 to 4400 J/m^3^) of the EDPC5**Bi5** adhesive film. **Be** and **Ba**, both of which have an aromatic ring, also substantially increased the mechanics of the cured adhesive films. In particular, **Ba** was able to attack the epoxide and hydroxyl moieties of the epoxy and phenoxy resins, thus reducing the compatibilizing effect. In contrast, **Ha** and **Sa**, neither of which has an aromatic ring, represented relatively low values. In particular, the mechanical properties of EDPC5**Sa5** were lower than those of EDPC5 without **Sa** because the aliphatic chain of **Sa** is considerably longer, thereby losing the compatibilizing effect. Despite the highest compatibilizing effect of **Bi**, only additives with acidic groups, such as **Ba**, can be utilized for the electrical interconnections in semiconductor packaging. Figure S5 shows that the compatibilizing agents without acidic groups (**Be** and **Bi**) did not achieve wetting of the solder balls on the Cu pads. Among the acidic additives, **Ba** showed the best mechanical properties and thus **Ba** was primarily selected for the rest of the tests in this study.

**Figure 7:**
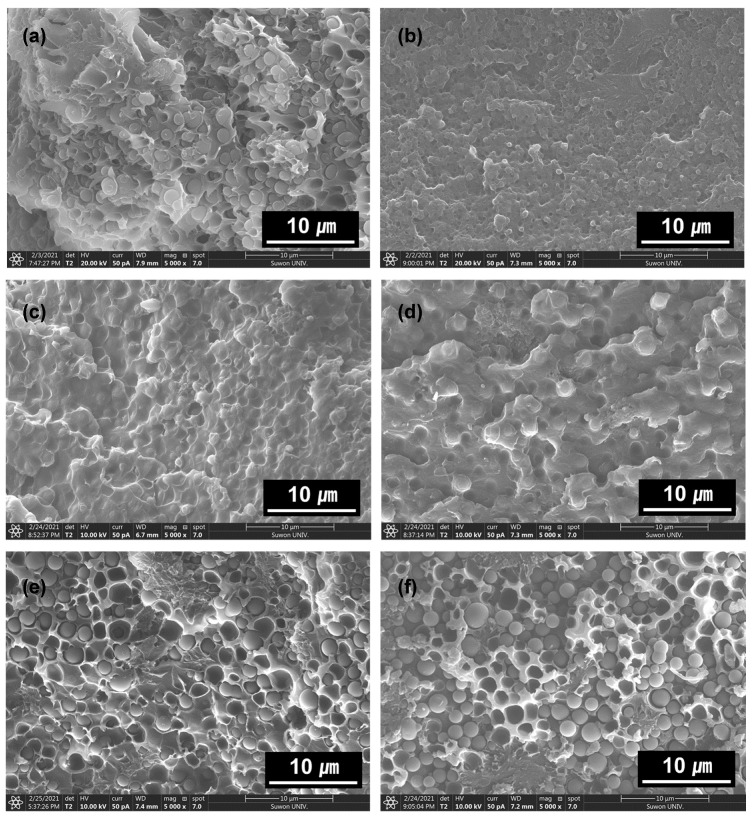
SEM images of the fractured cured adhesive film: (**a**) EDPC5, (**b**) EDPC5**Ba5**, (**c**) EDPC5**Be5**, (**d**) EDPC5**Bi5**, (**e**) EDPC5**Ha5**, and (**f**) EDPC5**Sa5**.

Similar to the correlation between the mechanical properties and morphological studies of EDPC, EDPM, and EDPS with **Ba**, the SEM images of EDPC5 with different additives also confirmed the origins of the mechanical properties. EDPC5**Ba5**, EDPC5**Be5**, and EDPC5**Bi5** exhibited the compatibilized morphologies, whereas the additives without an aromatic ring (**Ha** and **Sa**) showed the phase-separated domains of polycarbonate in the epoxy and phenoxy matrices, compared with EDPC5 without an additive, as shown in Figure 7. Thus, the phase separation between the domains and matrices hindered the mechanical properties of the cured hybrid adhesive films.

**Figure 8:**
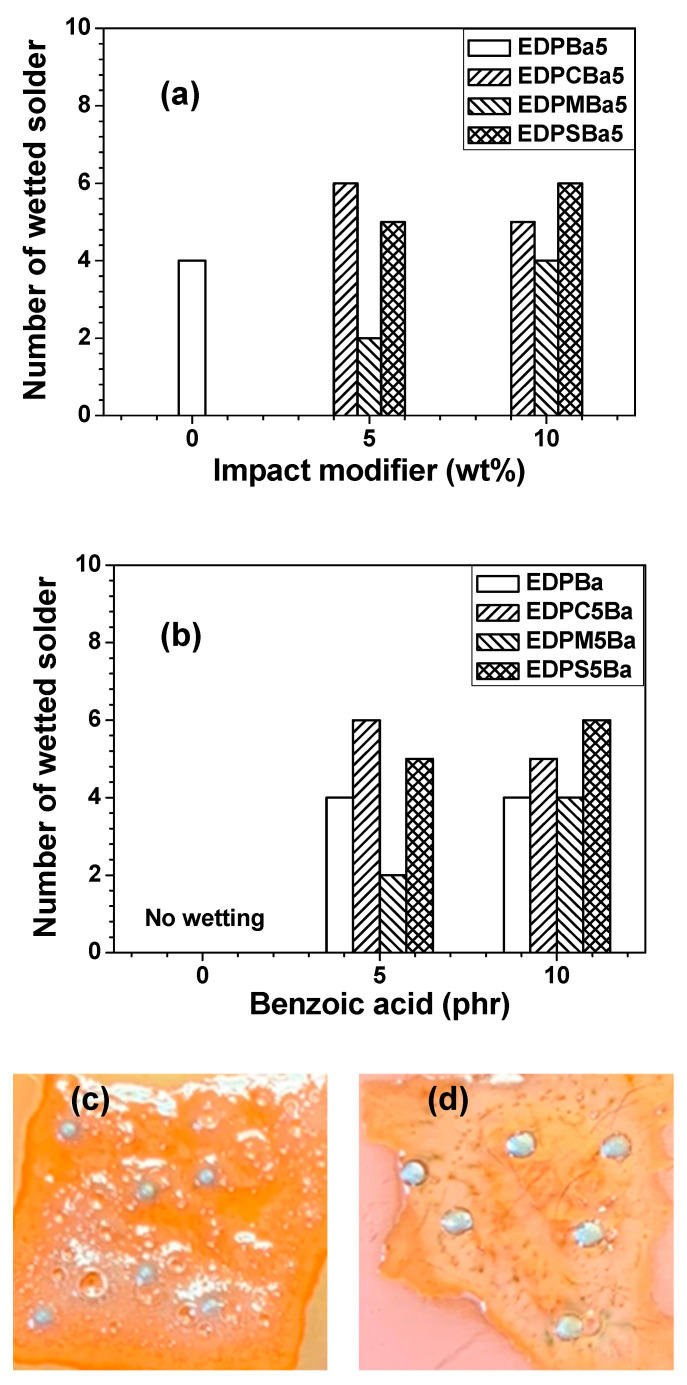
The wetting tests using SAC305 solder balls on Cu substrates: (**a**) number of wetted SAC305 balls as a function of amorphous polymer additive content, (**b**) number of wetted SAC305 balls as a function of benzoic acid, (**c**) EDPC5 and (**d**) EDPC5**Ba5**.

The deoxidizing (fluxing; removing the metal oxide layers on the metal solder surfaces) capability [11,15,18] can be achieved by hydroxyl, amino and acidic moieties, organoamine hydrochlorides, rosin-based fluxes, and inorganic acids of hybrid adhesive films. In this study, acidic groups were utilized for the fluxing capability. The fluxing capability resulted in the wetting behavior. The electrical interconnections in semiconductor packaging require good wettability of Sn-based metal solders. Figure 8 shows the wettability of the hybrid adhesive films. The SAC305 solder balls in the adhesive film containing no benzoic acid remained non-wetted owing to the absence of acidic groups, as shown in Figure 8b,c and Figure S6. The incorporation of additive binders into adhesive films rarely influenced the wettability, except for the EDPM5 series, probably because the benzoic acid coalesced in the uncompatibilized matrices.

**Figure 9:**
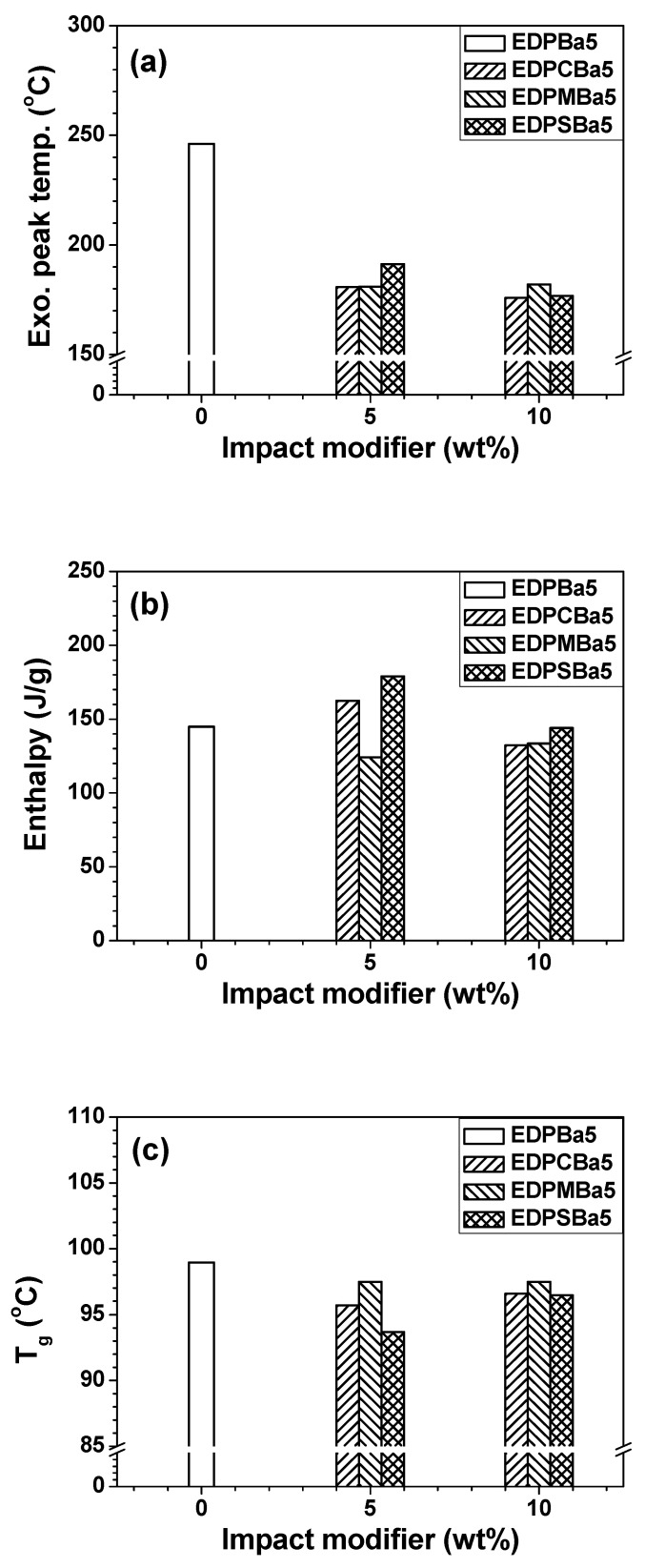
DSC results of adhesive films with different binders as a function of binder content: (**a**) exothermic reaction peak temperature, (**b**) reaction enthalpy, and (**c**) glass transition temperature of the cured adhesive films during the second scan.

The curing behaviors, exothermic reaction peak temperature and enthalpy of the uncured adhesive films, and glass transition temperature (*T_g_*) of the cured adhesive films were examined by DSC as shown in Figure 9, Figures S7 and S8. The incorporation of amorphous polymer additives into the adhesive films slightly influenced the curing behaviors, exothermic peak temperatures, and reaction enthalpy. The infiltration of additives slightly reduced the *T_g_* values of the cured adhesive films because the additives decreased the compatibility between the matrices and additive domains. The presence of SAC305 in the EDPC5**Ba5** adhesive film only altered the curing behavior to a small degree, as shown in Figure S9.

**Figure 10:**
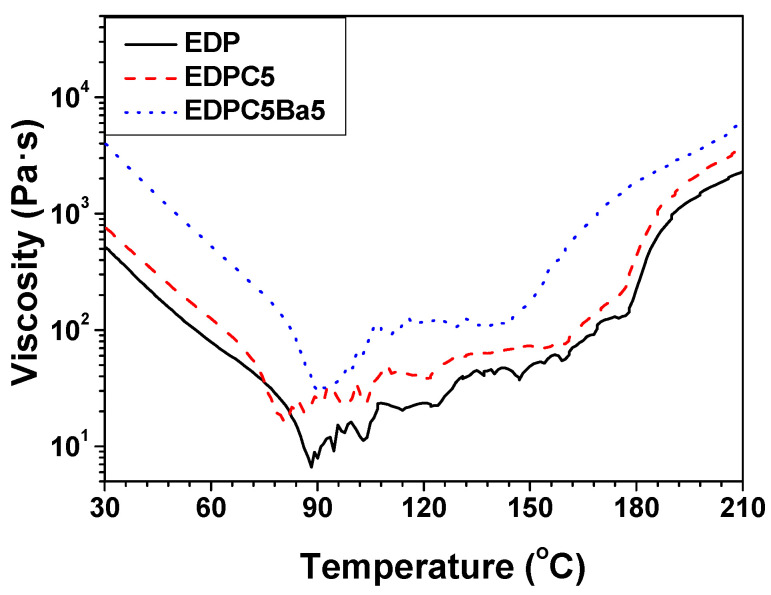
Viscosity of adhesive films as a function of temperature.

Chemorheological behaviors were examined by rheometry to understand the correlation between the curing reaction and viscosity. The viscosities of the adhesive films commenced to decrease at the initial stage of heating because the curing reaction rarely occurred until ca. 90 °C, as shown in Figure 10. Beyond this point, the curing reaction aggressively occurred and dominated the thermally induced softening behaviors, thereby increasing the viscosity. The EDP adhesive film showed the lowest viscosity over the entire temperature range and the latest increment in viscosity due to the high curing reaction temperature, which is in good agreement with the DSC curves in Figure S8. The incorporation of polycarbonate additive into the adhesive film increased the viscosity due to the high molecular weight of polycarbonate (low melt flow index) and rarely influenced the curing behaviors because of the non-reactivity of polycarbonate with hydroxyl or epoxide groups. The viscosity of EDPC5**Ba5** was higher than that of the other adhesive films, and the curing reaction temperature decreased owing to the reactive moieties of **Ba**. The initial curing temperatures measured by DSC were lower than those measured by rheometry because the sample content for DSC was substantially lower than that in the rheometry test and the generated heat detection is commonly faster than the mechanical responses in, for example, dynamic mechanical analysis, thermal-mechanical analysis, and rheometry [25].

**Figure 11:**
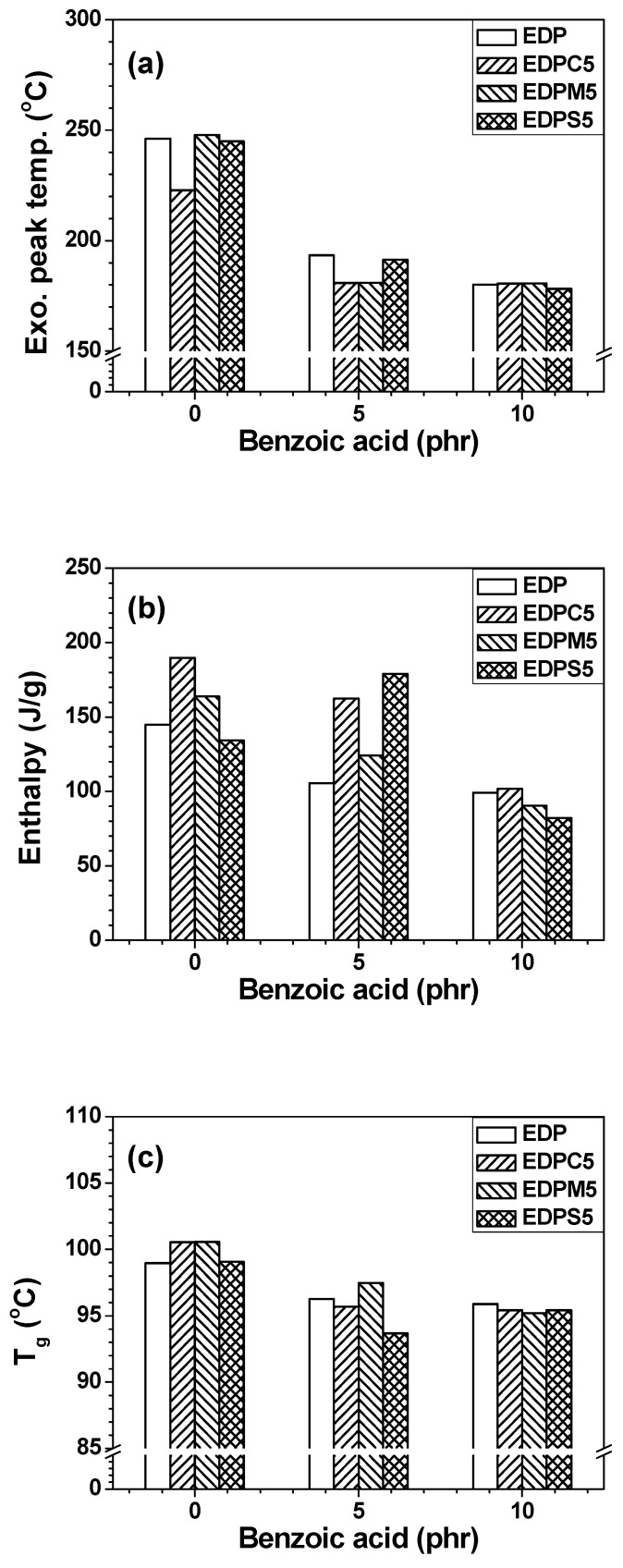
DSC results of adhesive films with different binders as a function of **Ba** loading: (**a**) exothermic reaction peak temperature, (**b**) reaction enthalpy during the first heating scan, and (**c**) *T_g_* of the cured adhesive films during the second heating scan.

The effects of **Ba** on curing behaviors, exothermic reaction peak temperature and enthalpy of the uncured adhesive films and *T_g_* of the cured adhesive films were also investigated by DSC (Figure 11). The exothermic reaction peak temperatures of EDP, EDPC5, EDPM5, and EDPS5 adhesive films decreased with an increasing **Ba** concentration owing to the curing and catalytic reactivity of **Ba** in the curable adhesive films. The reaction enthalpy slightly changed at 5 phr **Ba**. Above 10 phr **Ba,** the enthalpy substantially decreased because of excessive **Ba** content, which complicated the reaction mechanism and reduced the cross-linking density due to the mono-reactive acidic moiety of **Ba**. Similarly, the *T_g_* values were also reduced by the incorporation of **Ba** owing to the **Ba**-induced decrease in cross-linking density. **Ba** is necessary for the deoxidization of Sn-based metal solders that leads to electrical interconnections in the semiconductor packaging. The **Ba** incorporation into the hybrid adhesive films resulted in deoxidization and reactivity changes.

**Figure 12:**
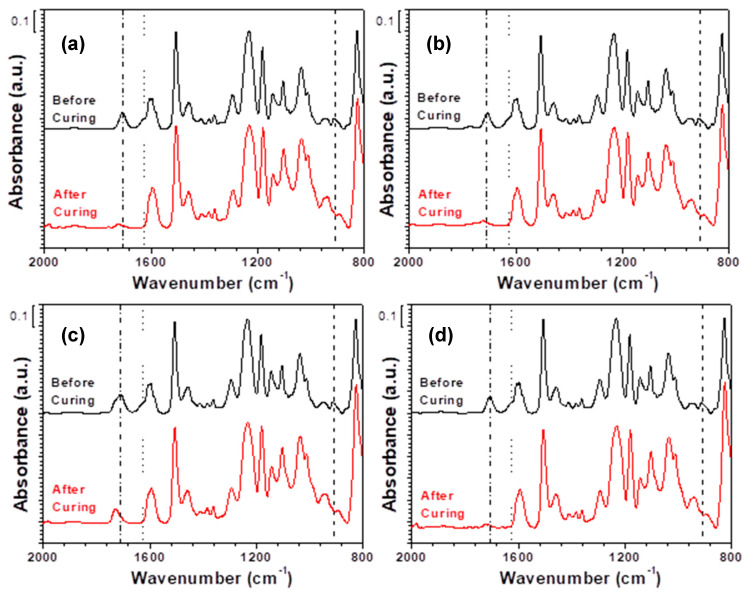
FTIR ATR spectra: (**a**) EDP**Ba5**, (**b**) EDPC5**Ba5**, (**c**) EDPM5**Ba5**, and (**d**) EDPS5**Ba5**. (a.u. means arbitrary units).

Spectroscopic analyses [26,27], such as through FTIR spectroscopy, are extensively utilized to detect the chemical structures of epoxides, acids, and amines. Figure 12 shows the ATR FTIR spectra of EDP**Ba5**, EDPC5**Ba5**, EDPM5**Ba5**, and EDPS5**Ba5** before and after curing. The peaks for epoxide (913 cm^−1^), acid (1710 cm^−1^), and amine (1625 cm^−1^) of the hybrid adhesive films substantially decreased after curing. This indicates that not only typical curable substances but also acidic moieties participated in the curing reactions. The multifunctional **Ba** additive acts as a compatibilizing, deoxidizing, and curing agent in the hybrid adhesive films. This result is in good agreement with the curing, mechanical, thermal, and rheological properties investigated in this study. The acidic moieties in EDPM5**Ba5** were less consumed compared with EDP**Ba5**, EDPC5**Ba5**, and EDPS5**Ba5**, because PMMA might impede the curing reaction between **Ba** and epoxide. The peak at 1600 cm^−1^ is ascribed to the conjugation of the C=C bond with the C=O bond in **Ba** and partially overlaps with the peak at 1625 cm^−1^ for N-H bending.

**Figure 13:**
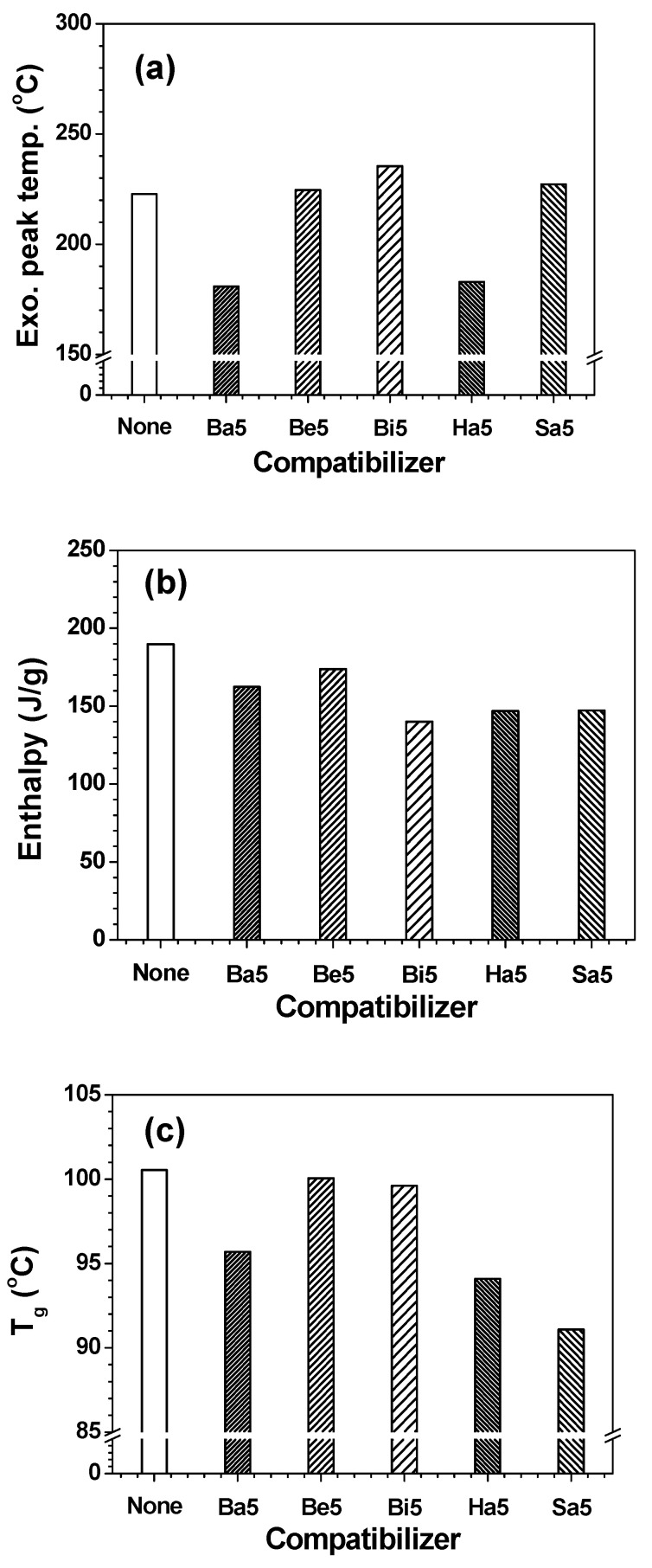
DSC results of EDPC5 with different compatibilizers at a loading of 5 phr: (**a**) exothermic reaction peak temperature, (**b**) reaction enthalpy during the first heating scan, and (**c**) *T_g_* of the cured adhesive films during the second heating scan.

The effects of various compatibilizers (**Ba**, **Be**, **Bi**, **Ha**, and **Sa**) on the curing behaviors, exothermic reaction peak temperature and enthalpy of the uncured adhesive films, and *T_g_* of the cured adhesive films were examined, as shown in Figure 13 and Figure S10. The exothermic reaction peak temperatures were barely influenced by the incorporation of **Be5**, **Bi5**, and **Sa5**, whereas they were reduced by **Ba5** and **Ha5** because of the curing reaction of the mono-reactive groups with epoxides. The reaction enthalpies of the adhesive films were slightly decreased by the additive incorporation due to the curing reaction of mono-reactive groups and residual unreactive molecules. As the concentration of unreactive molecules increased, the proportion of the reactive moieties decreased, thereby decreasing the probability of exothermic reactions. The additives in the adhesive films decreased the *T_g_s* of the cured adhesive films, owing to the mono-reactive groups and residual unreactive molecules, which resulted in plasticization effects. The 5 phr **Sa** significantly decreased the *T_g_* of the cured EDPC5**Sa5** adhesive film because of its long aliphatic chain.

## 4. Conclusions

We fabricated the robust hybrid adhesive films with deoxidizing and curing capabilities for electrical interconnections and adhesion. We also examined the effects of additive binders (PC, PMMA, and PS) and compatibilizing agents on the curing, deoxidizing, mechanical, thermal and rheological properties. Small amounts (5 phr) of polymer binders and benzene-containing compatibilizers enhanced the various properties, such as mechanical properties. The EDPC5**Ba5** composition was determined to be the most effective adhesive film for deoxidizing curable capabilities and film formation, despite the highest compatibilizing effects of **Be** and **Bi**, due to the absence of acidic moieties. These fabricated adhesive films can be utilized for electronic device adhesion (e.g., semiconductor packaging) and the effects of additives and compatibilizing agents can be used for various blending applications.

## Figures and Tables

**Table 1:** Full names, abbreviations, and sample names of components.

Name	Epoxy	DDS	Phenoxy	PC	PMMA	PS
Sample name	E	D	P	C	M	S
Name	Benzoic acid	Benzene	Biphneyl	Hexanoic acid	Stearic acid	
Sample name	**Ba**	**Be**	**Bi**	**Ha**	**Sa**	

## Data Availability

The data are not publicly available due to technical or time limitations at this time.

## Supplementary Materials

The following are available online at https://www.mdpi.com/article/10.3390/polym13071129/s1, Figure S1: Chemical structures of PMMA, PS, benzene, biphenyl, hexanoic acid and stearic acid, Figure S2: (a) Wetting test of solder ball on Cu plate and (b) bonding procedure between chip and substrate, Figure S3: Enlarged SEM images of fractured cured adhesive film with different polymer additives: (a) EDP, (b) EDP**Ba5**, (c) EDPC5, (d) EDPC5**Ba5**, (e) EDPM5, (f) EDPM5**Ba5**, (g) EDPS5, and (h) EDPS5**Ba5**, Figure S4: Adhesive films with different polycarbonate contents: (a) EDPC5**Ba5**, (b) EDPC10**Ba5**, (c) EDPC20**Ba5**, and (d) EDPC30**Ba5**, Figure S5: Wetting test of SAC305 solder ball on Cu plate: EDPC5 with various additives of 5 phr, Figure S6: Wetting test of SAC305 solder ball on Cu plate as a function of **Ba** concentration, Figure S7: DSC scans of hybrid adhesive films with different additive binders: (a) EDPC**Ba5**, (b) EDPM**Ba5**, and (c) EDPS**Ba5**, Figure S8: DSC scans of hybrid adhesive films with different additive binders: (a) EDP, (b) EDPC, (c) EDPM, and (d) EDPS, Figure S9: DSC scans: EDPC5**Ba5** with and without SAC305 solder balls at a heating rate of 10 °C/min, Figure S10: DSC scans of EDPC5 with different compatibilizers (**Ba**, **Be**, **Bi**, **Ha**, and **Sa**) at loading of 5 phr.
