# Supplementary material for: Impact Modifiers and Compatibilizers for Versatile Epoxy-Based Adhesive Films with Curing and Deoxidizing Capabilities
Source: Polymers (Basel). 2021 Apr 2;13(7):1129. doi: 10.3390/polym13071129 (PMC8037572; doi:10.3390/polym13071129)
Supplement: Supplementary file 1 [file polymers-13-01129-s001.pdf]

polymers

MDPI

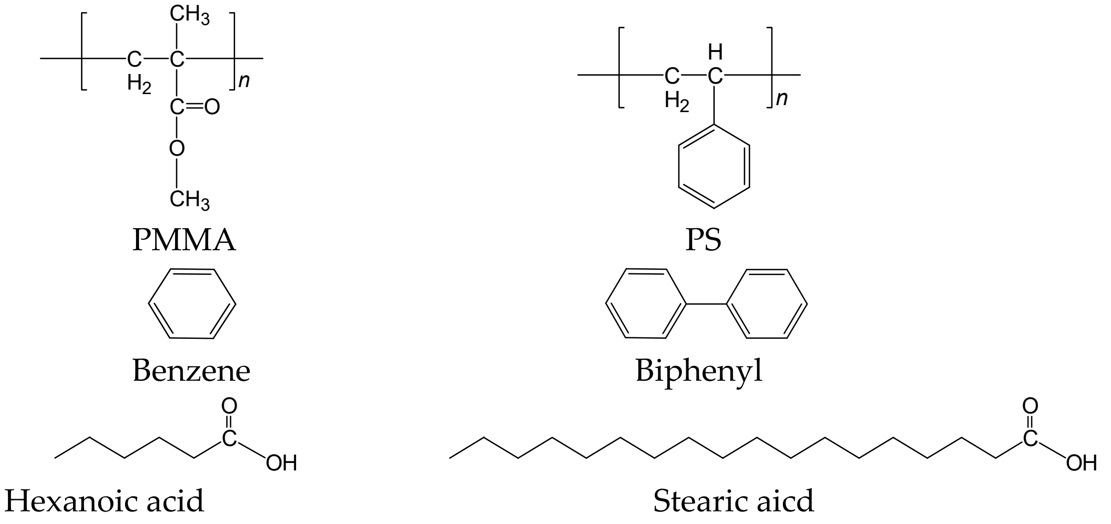

Figure S1. Chemical structures of PMMA, PS, benzene, biphenyl, hexanoic acid and stearic acid.

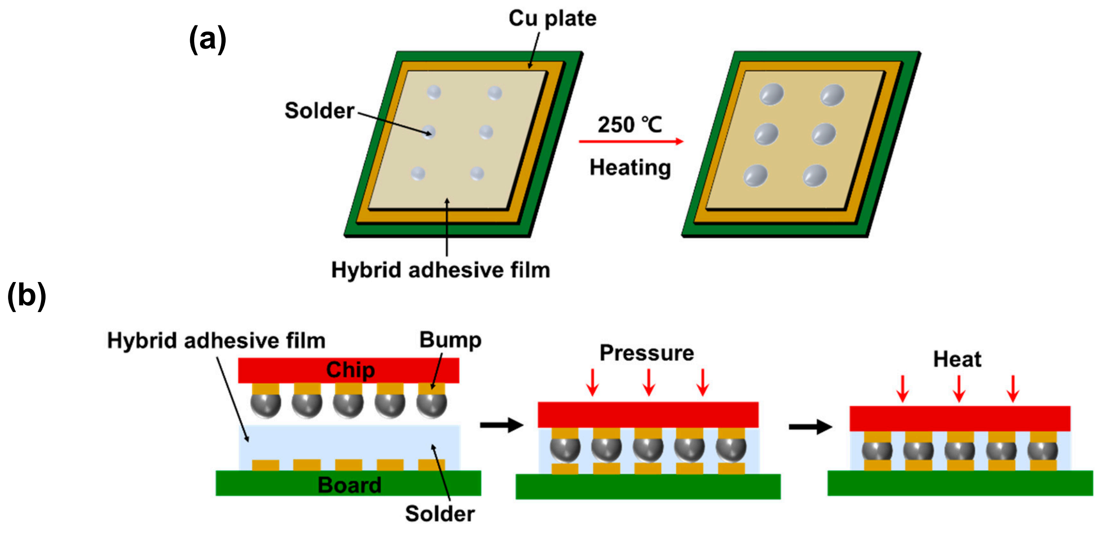

Figure S2. (**a**) Wetting test of solder ball on Cu plate and (**b**) bonding procedure between chip and substrate.

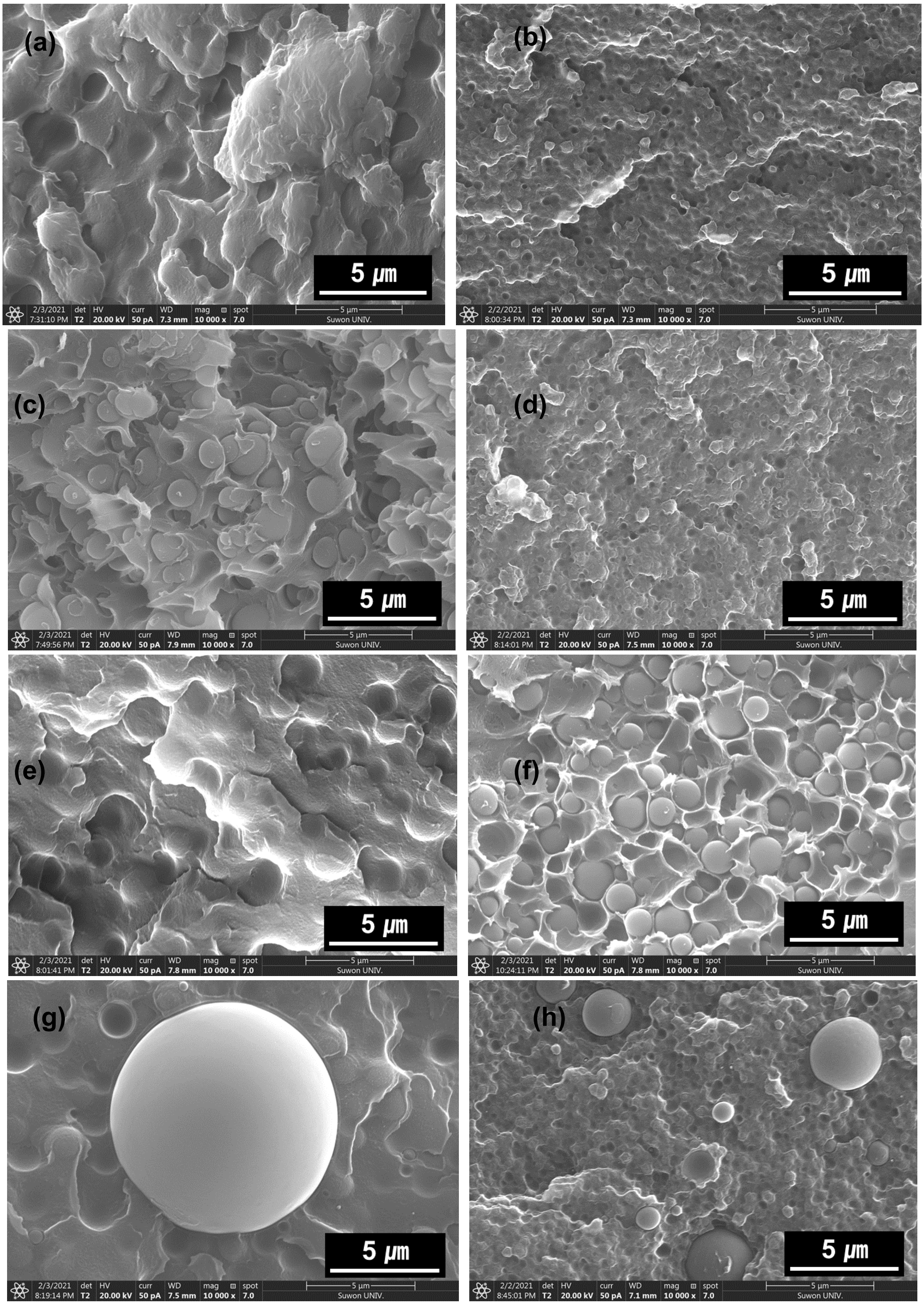

Figure 3. Enlarged SEM images of fractured cured adhesive film with different polymer additives: (**a**) EDP, (**b**) EDP**Ba5**, (**c**) EDPC5, (**d**) EDPC5**Ba5**, (**e**) EDPM5, (**f**) EDPM5**Ba5**, (**g**) EDPS5, and (**h**) EDPS5**Ba5**.

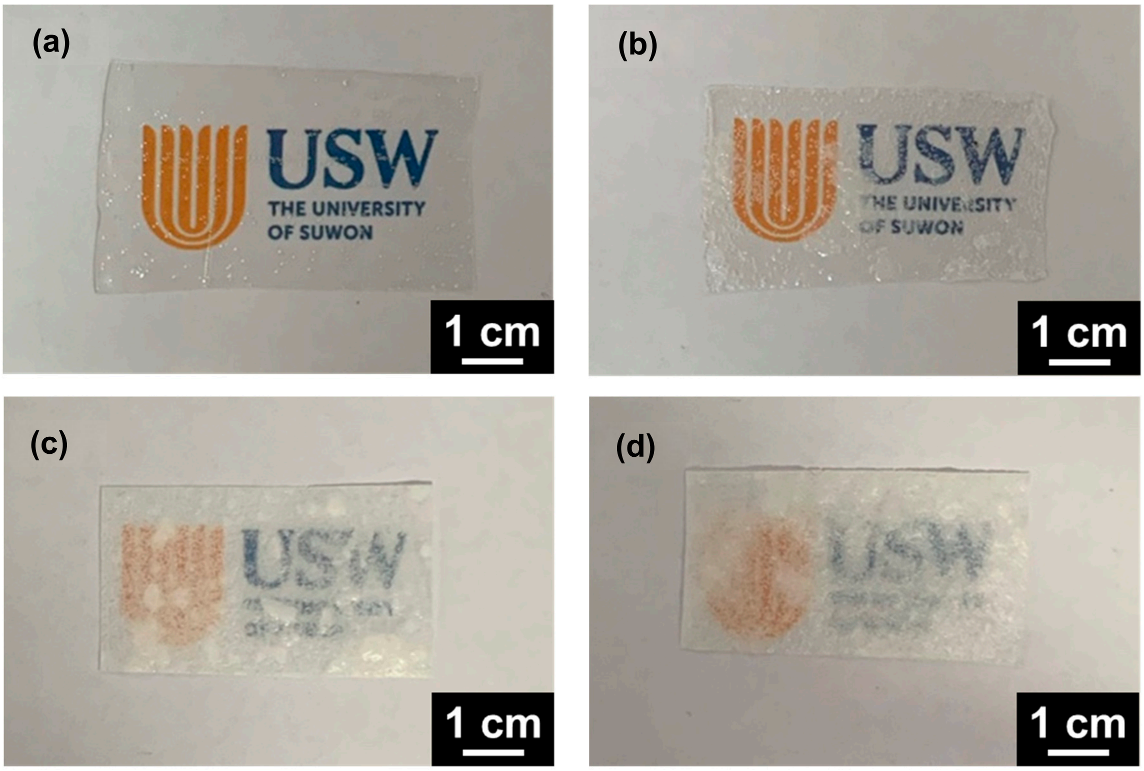

Figure S4. Adhesive films with different polycarbonate contents: (**a**) EDPC5**Ba5**, (**b**) EDPC10**Ba5**, (**c**) EDPC20**Ba5**, and (**d**) EDPC30**Ba5**.

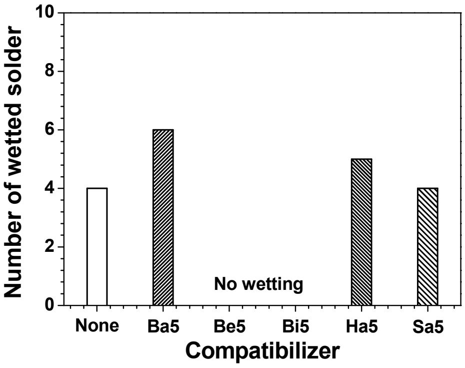

Figure S5. Wetting test of SAC305 solder ball on Cu plate: EDPC5 with various additives of 5 phr.

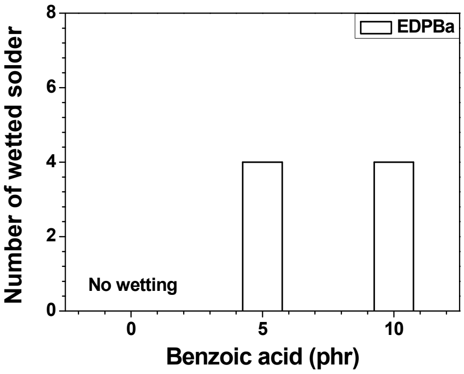

Figure S6. Wetting test of SAC305 solder ball on Cu plate as a function of **Ba** concentration.

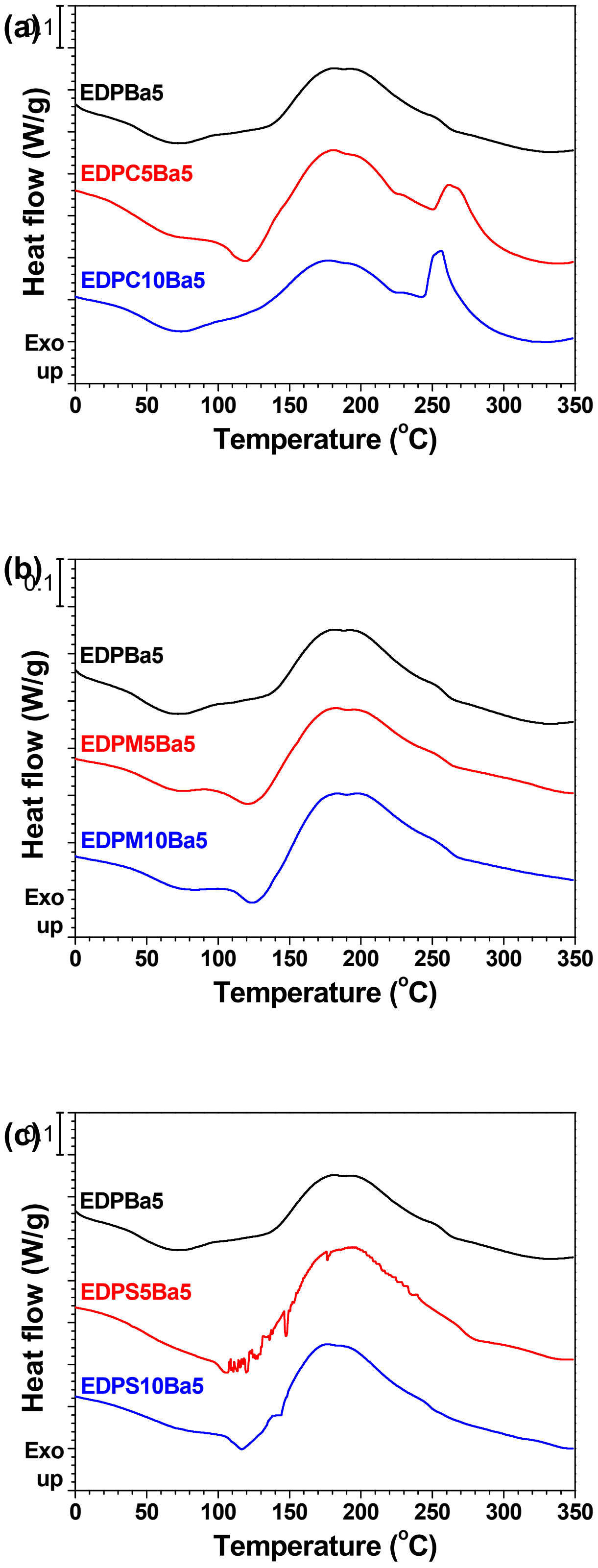

Figure S7. DSC scans of hybrid adhesive films with different additive binders: (**a**) EDPC**Ba5**, (**b**) EDPM**Ba5**, and (**c**) EDPS**Ba5**.

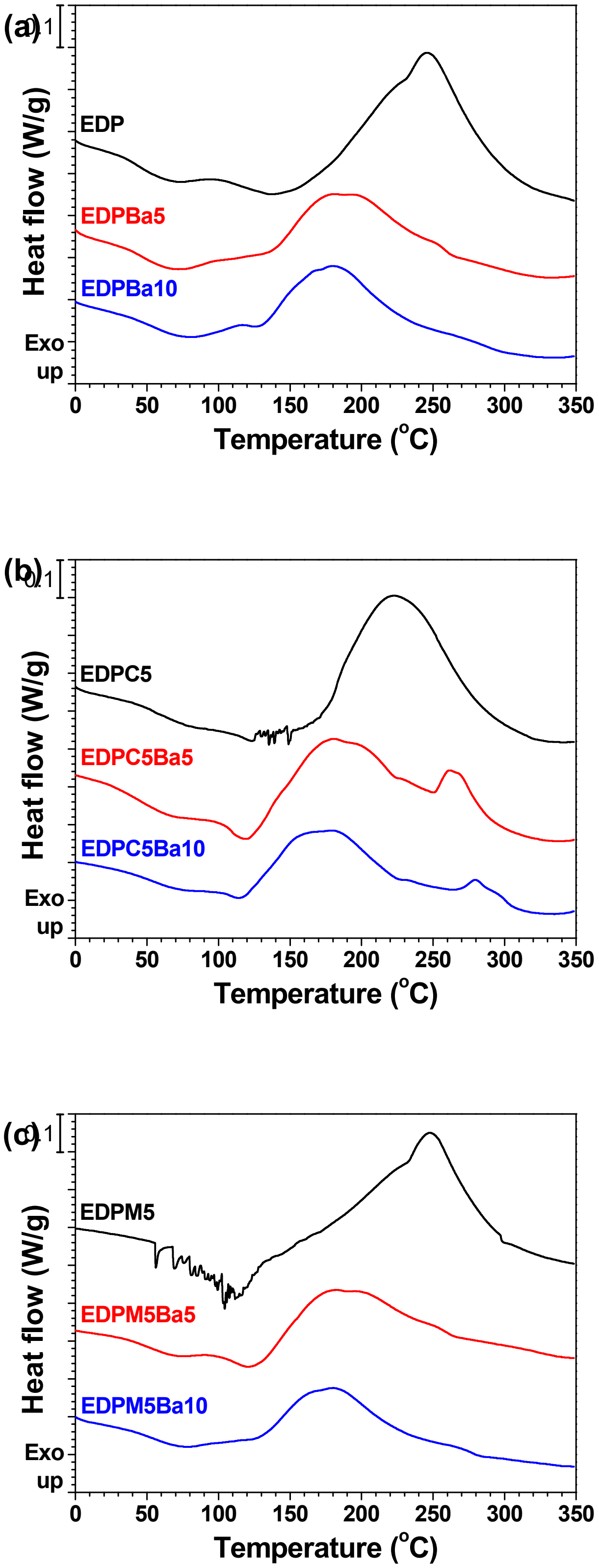
(a)
0.1
Heat flow (W/g)
EDP
EDPBa5
EDPBa10
Exo
up
0 50 100 150 200 250 300 350
Temperature (°C)
(b)
0.1
Heat flow (W/g)
EDPC5
EDPC5Ba5
EDPC5Ba10
Exo
up
0 50 100 150 200 250 300 350
Temperature (°C)
(c)
0.1
Heat flow (W/g)
EDPM5
EDPM5Ba5
EDPM5Ba10
Exo
up
0 50 100 150 200 250 300 350
Temperature (°C)

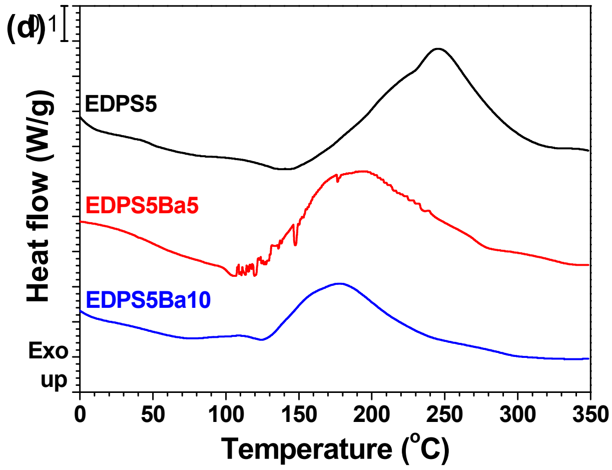

Figure S8. DSC scans of hybrid adhesive films with different additive binders: (**a**) EDP, (**b**) EDPC, (**c**) EDPM, and (**d**) EDPS.

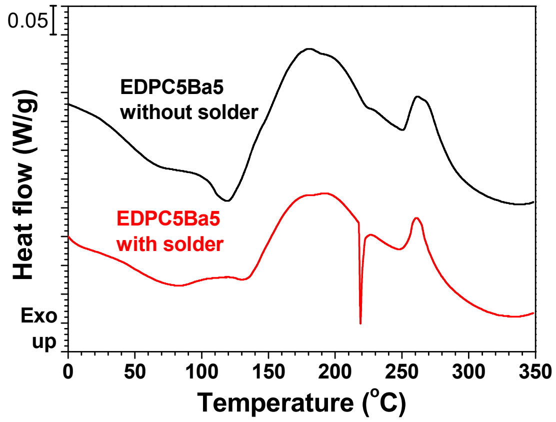

Figure S9. DSC scans: EDPC5**Ba5** with and without SAC305 solder balls at a heating rate of 10 °C/min.

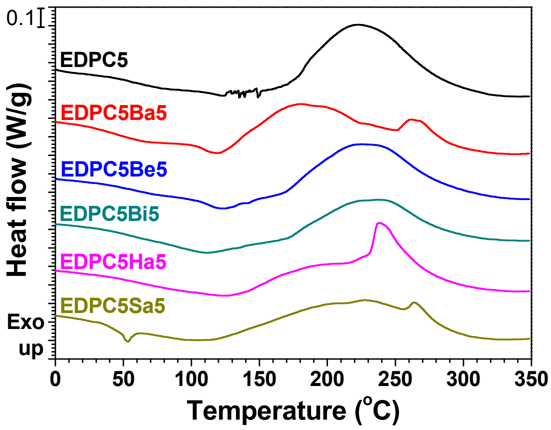

Figure 10. DSC scans of EDPC5 with different compatibilizers (**Ba**, **Be**, **Bi**, **Ha**, and **Sa**) at loading of 5 phr.
